# Supplementary material for: Barriers of West African women scientists in their research and academic careers: A qualitative research
Source: PLoS One. 2022 Mar 30;17(3):e0265413. doi: 10.1371/journal.pone.0265413 (PMC8967005; doi:10.1371/journal.pone.0265413)
Supplement: Attachment — Submitted filename: Responses to Reviewers.docx [file pone.0265413.s001.docx]

Responses to Reviewers

Reviewers 1 :

The discussion was reinforced from a theoretical perspective. Fagenson's theory was used as a theory framework. The literature suggested by reviewer 1 has been exploited and integrated as being relevant to the work done in our introduction.

Lines 27 and 28: the sentence referring to a part of the methodology in the abstract has been removed. It is found in the methodology

Page 9, line 47: the acronym STEM has been introduced before reference 2

Reviewer 2

1- The context was enhanced with data on African academies and their gender policies. Previous research has been integrated into the study.
2- Previous studies concerning the themes have been included in the study's theorical and analitycal framework. The introduction has been improved by the latter allowing a better understanding of the research results.
3- A better contextualization of African research institutes was done. Policies regarding gender equality in African research spaces, African institutes and the countries concerned by the study were made.
4- The methodology section has been improved in terms of form. The subdivisions (study design, study population, sampling and data collection and analysis) and have been removed
5- The introduction has been improved to better support the results and conclusions of the study

Editor’s comments

1. We have respected PLOS ONE’s style requirements
2. The participation of researchers from different West African countries in this research is governed by the provisions of the West African Network of National Ethics Committees of Research for Health (WANEC), of which the National Ethics Committee of Research for Health of Senegal is a member
3. We include more information on the number of interviewers and their characteristics
4. Reference list are reviewed
